# hsa_circRNA_000166 Facilitated Cell Growth and Limited Apoptosis through Targeting miR-326/LASP1 Axis in Colorectal Cancer

**DOI:** 10.1155/2020/8834359

**Published:** 2020-12-09

**Authors:** Qin Hao, Zhongtao Zhang

**Affiliations:** ^1^Department of General Surgery, Beijing Friendship Hospital, Capital Medical University, Beijing 100050, China; ^2^National Clinical Research Center of Digestive Diseases, 100050 Beijing, China; ^3^Department of Gastrointestinal Surgery, Affiliated Hospital, Inner Mongolia Medical University, 010051 Hohhot, China

## Abstract

Circular RNAs (circRNAs) belong to noncoding RNAs and are widely expressed in a variety of cell species, including cancers. However, the function and mechanism of circRNAs in colorectal cancer (CRC) has not been well investigated. Here, we firstly downloaded and analyzed the circRNA expression profile of CRC from the Gene Expression Omnibus (GEO) database. And we identified 181 differentially expressed circRNAs between 10 pairs of CRC and adjacent normal tissues. Interestingly, we observed that the expression of hsa_circRNA_000166 was the top increased among these circRNAs. Then, we confirmed an upregulation of hsa_circRNA_000166 in CRC tissues and cell lines and observed that higher expression of hsa_circRNA_000166 was associated with poor 5-year survival rate of patients with CRC. Next, we investigated the function of hsa_circRNA_000166 during CRC progression by knocking down its expression. Cell growth and apoptosis assay revealed that hsa_circRNA_000166 regulated the cell growth and apoptosis in CRC cell lines. Furthermore, we identified that hsa_circRNA_000166 targeted the miR-326/LASP1 pathway using bioinformatics analysis and luciferase reporter assay. Finally, suppression of miR-326 or overexpression of LASP1 could sufficiently rescue the aberrant cell growth and apoptosis in CRC cell lines. Taken together, our results indicated that downregulation of hsa_circRNA_000166 inhibited the cell growth and facilitated apoptosis during CRC development by sponging the miR-326/LASP1 pathway.

## 1. Introduction

Colorectal cancer (CRC) is one of the malignant cancers with the highest incidence and the fourth leading cause of cancer-related mortality around the world [1]. Recently, according to changes in dietary patterns and physical activity, CRC has dramatically increased in China [2]. Despite the advanced surgery technologies and medicine treatments that have been applied in CRC treatment, the survival rate of patients with CRC still remains unsatisfactory [3–7]. Hence, there is an urgent call for new breakthrough regarding the mechanisms underlying CRC progression.

Circular RNAs (circRNAs), a subgroup of noncoding RNAs, have a crucial role in regulating gene expression and function in distinct biological processes [8–12]. Different with linear RNAs, circRNAs have covalently closed continuous loops, which result in increased stability [13, 14]. Multiple evidences have demonstrated that the expression of circRNAs is aberrant in various cancers [15–19]. In general, circRNAs mainly served as upstream regulator to control the expression of microRNA (miRNA) during tumorigenesis [16, 20–22]. Previous studies have shown that circRNAs have an essential role in CRC progression by the regulation of multiple miRNAs [18, 23, 24]. However, the function of dysregulated circRNAs during the development of CRC remains to be further elucidated.

In our study, we downloaded the circRNA expression profile of CRC from the Gene Expression Omnibus (GEO) database. After analysis, we identified 181 differentially expressed circRNAs and observed a top overexpression of hsa_circRNA_000166 among them. Subsequently, we revealed the ectopic expression of hsa_circRNA_000166 in CRC tissues and cell lines, which was associated with the 5-year survival rate of patients with CRC. Next, we knocked down the expression of hsa_circRNA_000166 using small interfering RNA (siRNA) to explore the potential roles of hsa_circRNA_000166 during CRC progression. We observed that hsa_circRNA_000166 inhibited the cell proliferation and promoted apoptosis in CRC cell lines. Moreover, we evidenced that hsa_circRNA_000166 directly regulated the miR-326/LASP1 pathway and the aberrant cell growth and apoptosis could be rescued after forced expression of miR-326 in CRC cell lines. In summary, our findings revealed that hsa_circRNA_000166 promoted the cell growth and repressed apoptosis via inducing the miR-326/LASP1 pathway during CRC tumorigenesis, which might be a promising candidate for diagnostic and therapeutic application in CRC treatment.

## 2. Materials and Methods

### 2.1. Tissue Collection

The CRC tissues and adjacent normal colon tissues were obtained from Inner Mongolia Medical University Affiliated Hospital between 2015 and 2018. Totally, 40 pairs of tissues were analyzed in the study. Patients with CRC did not experience systemic treatment of chemotherapy or radiotherapy before surgery. All of the patients had got the written informed consent. The study followed the ethics committee of Inner Mongolia Medical University Affiliated Hospital guidance. All specimens were stored at −80°C until use.

### 2.2. Cell Culture

We cultured CRC cell lines SW1116, DLD-1, HCT116, SW480, and SW620 and human normal colonic epithelial cells HCoEpiC in minimum essential medium (MEM) (Gibco, 41500034) with 1% Glutamax (Invitrogen, 35050-061), 1% Nonessential Amino Acids, 100x (Invitrogen, 11140-050), and 10% fetal bovine serum (FBS). In a humidified atmosphere containing 5% CO_2_ at 37°C, the incubation of the cell lines mentioned above was performed. We purchased the cell lines from the Institute of Biochemistry and Cell Biology at the Chinese Academy of Sciences (Shanghai, China).

### 2.3. Microarray Datasets

Gene Expression Omnibus (GEO) (https://www.ncbi.nlm.nih.gov/geo/), a publicly available genomics database, is queried for all datasets. We downloaded the dataset of CRC, which was the circRNA expression profile from GEO. The selected dataset was in accordance with the following criteria. (1) They employed CRC tissue samples. (2) They took the adjacent normal tissues as control. (3) They utilized information on technology and platform.

### 2.4. Quantitative Real-Time PCR Assay

Total RNAs from tissues or cultured cells were isolated using TRIzol reagent (Invitrogen, Carlsbad, CA), following the manufacturer's instructions. For quantitative real-time PCR (qRT-PCR), the reverse transcription kit (Takara, Dalian, China) was used to reverse transcribe total RNA into cDNA according to the manufacturer's protocol, while a stem-loop RT-qPCR method was used to generate miRNAs. qRT-PCR was conducted in ABI StepOnePlus™ real-time PCR system (Applied Biosystems, Foster City, USA). U6 and GAPDH were applied as internal controls. The gene-specific primers are listed in Table 1.

### 2.5. Plasmid and Transfection

The siRNA-negative control (NC), siRNA-1, siRNA-2, siRNA-3, miR-NC, miR-326 mimics, miR-326 inhibitor (miR-326 I), hsa_circRNA_000166 wild-type (WT) plasmid, hsa_circRNA_000166 mutant (Mut) plasmid, LASP1 wild-type (WT) plasmid, mutant (Mut) plasmid, and LASP1 overexpression plasmid were constructed by GenePharma (Shanghai, China). According to the manufacturer's instructions, we transfected the plasmids into HCT116 and SW480 cells using the Lipofectamine 2000 Transfection Reagent (Invitrogen).

### 2.6. Cell Counting Kit-8 Assay

The Cell Counting Kit-8 (CCK-8) assay was used to detect cell growth of HCT116 and SW480 cells. Each group was incubated with a density of 104 cells in 96-well plates. Cells in each well were incubated which lasted for 2 h at 1, 2, and 3 days with CCK-8 reagent (Dojindo, Japan). We measured the optical density at 450 nm using an automatic microplate reader (Synergy4; BioTek).

### 2.7. Colony Formation Assay

We seeded the transfected cells into 6-well plates and cultured for 14 days and then fixed the cells with methanol and stained them with 0.5% crystal violet (Beyotime Biotechnology) for 30 min. Colonies with more than 10 cells were counted under a light microscope.

### 2.8. Flow Cytometric Assay

For apoptosis detection, the HCT116 and SW480 cells were transfected with different plasmids for 24 hours (h) before collection. Then, we used an Annexin V-FITC/PI apoptosis detection kit (Invitrogen) to label the HCT116 and SW480 cells with Annexin V and PI. Flow cytometry (FACScan; BD Biosciences) was used to detect and analyze the fluorescence (FL1) and red fluorescence (FL2).

### 2.9. Target Prediction

We obtained the sequence of hsa_circRNA_000166 from circBase (http://www.circbase.org). starBase v2.0 (http://starbase.sysu.edu.cn) and CircInteractome (https://circinteractome.nia.nih.gov) were utilized to predict the binding sites between hsa_circRNA_000166 and miRNAs.

### 2.10. Dual Luciferase Reporter Assay

We constructed pGL3-promoter driven miR-326 luciferase reporter containing the binding site for hsa_circRNA_000166. And then, we used Lipofectamine 2000 (Invitrogen) to transfect the luciferase reporter with hsa_circRNA_000166 WT with wild binding site (CCCAGAG) and hsa_circRNA_000166 Mut with mutant binding site (GGGUCCU) into the HCT116 and SW480 cells. The firefly luciferase activity was detected at 48 h after transfection using the Dual Luciferase Reporter Assay system (Promega).

### 2.11. Western Blot Assay

For protein isolation after transfection, cells were lysed in the RIPA buffer (Beyotime, China). The SDS-PAGE gel assay was utilized to separate the proteins, and then, we transferred the separated proteins onto nitrocellulose membranes (GE Healthcare). Primary antibodies were used to incubate the membranes overnight at 4°C, followed by washing the membranes for 5 times using phosphate-buffered saline supplemented with Tween 20 (PBST). Subsequently, the corresponding horseradish peroxidase-conjugated secondary antibodies (Santa Cruz) were used to incubate the membranes for 2 h at room temperature. Finally, the SuperSignal West Femto kit (Pierce, Rockford, IL) was utilized to bring the bands on the membranes into visualization in the final. The primary antibodies and secondary antibody were used as follows: rabbit anti-LASP1 (1 : 2000, Abcam, ab117806), rabbit anti-GAPDH (1 : 5000, Abcam, ab181602), and goat anti-rabbit IgG H&L (HRP) (1 : 1500, Abcam, ab205718). We used GAPDH as the endogenous control in this assay.

### 2.12. Statistical Analysis

For significant difference analysis, GraphPad Prism 5.0 software was used to perform all the data. All results were analyzed using the two-tailed Student *t*-test and shown as the mean ± SD; ^∗^*P* < 0.05, ^∗∗^*P* < 0.01, and ^∗∗∗^*P* < 0.001.

## 3. Results

### 3.1. Microarray Data Information and DEG Analysis in Colorectal Cancer

**Figure 1:**
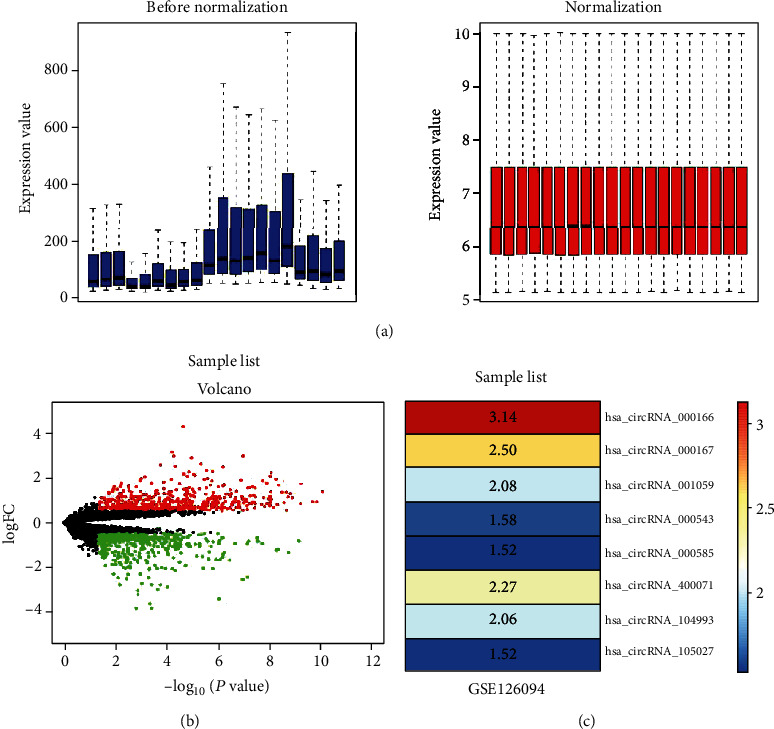
Microarray data information and DEG analysis in colorectal cancer. (a) The standardization of GSE126094 data. The data before normalization were displayed as the blue bar, while the normalized data were shown as the red bar. (b) The volcano plots of GSE126094 data. The red and green points, respectively, represented upregulated and downregulated genes screened on the basis of ∣fold change (FC) | >2.0 and a corrected *P* value < 0.05. Genes with no significant difference were shown as the black points. (c) The abscissa was defined as GEO ID, and the ordinate was defined as the gene name. The values in the box represent the log FC values. ANOVA test was used for statistics.

We downloaded the circRNA expression microarray dataset GSE126094 associated with colorectal cancer from the Gene Expression Omnibus (GEO) database and normalized (Figure 1(a)). Then, we screened the dataset to obtain differentially expressed genes (DEGs) using the limma package (∣log FC | >1 and FDR < 0.05). Volcano plots showed the differential expression of multiple circRNAs from the microarray dataset (Figure 1(b)). We obtained 181 DEGs from the GSE126094 dataset, including 74 upregulated circRNAs and 107 downregulated circRNAs. R-heat map software was used to draw a heat map of the top 8 upregulated circRNAs (Figure 1(c)). We found that the expression of hsa_circRNA_000166 was the highest among the upregulated circRNAs (Figure 1(c)), which suggested that hsa_circRNA_000166 might play a vital role during CRC progression.

### 3.2. Upregulation of hsa_circRNA_000166 in CRC Tissues and Cell Lines

**Figure 2:**
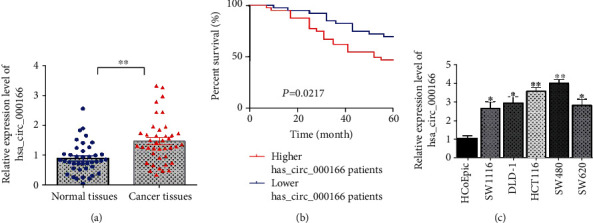
hsa_circRNA_000166 was upregulated in CRC tissues and cell lines. (a) qRT-PCR assay showed that the expression of hsa_circRNA_000166 was increased in CRC tissues (*n* = 40) compared with adjacent normal lung tissues (*n* = 40). (b) The Kaplan–Meier curve displayed 5-year survival rate of CRC patients with different hsa_circRNA_000166 expression levels (*n* = 40, *P* = 0.0217). (c) qRT-PCR assay showed an upregulation of hsa_circRNA_000166 in CRC cell lines compared with normal colonic cells. The Student *t*-test was used for statistics.

To figure out the potential function of hsa_circRNA_000166 during CRC development, we firstly detected the expression level of hsa_circRNA_000166 in CRC tissues and adjacent normal colonic tissues. And we observed the overexpression of hsa_circRNA_000166 in CRC tissues compared with the matched normal tissues using qRT-PCR assay (Figure 2(a)). Then, we divided the patients into two groups based on higher or lower hsa_circRNA_000166 expression in CRC tissues. The Kaplan–Meier survival curve displayed that patients with higher hsa_circRNA_000166 expression had a poor 5-year survival rate than the patients with lower hsa_circRNA_000166 expression (Figure 2(b)). Moreover, we also tested the expression of hsa_circRNA_000166 in CRC cells, such as SW1116, DLD-1, HCT116, SW480, and SW620, and human normal colonic epithelial cells HCoEpiC. Consistent with that in CRC tissues, we found the transcriptional level of hsa_circRNA_000166 was significantly increased in CRC cell lines compared to normal colonic cells (Figure 2(c)). Therefore, we verified the ectopic expression of hsa_circRNA_000166 in both CRC tissues and cell lines, which related to poor 5-year survival rate of CRC patients.

Downregulation of hsa_circRNA_000166 suppressed cell growth and promoted apoptosis in CRC cells.

**Figure 3:**
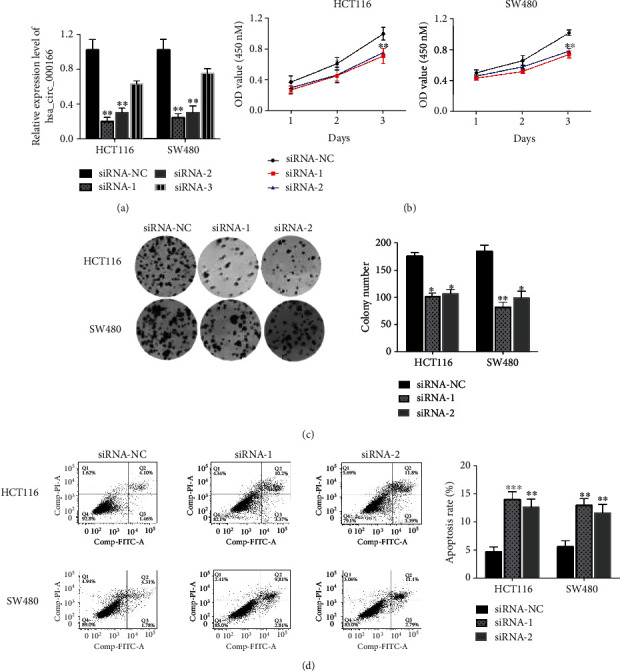
Decreased hsa_circRNA_000166 expression affected cell growth and apoptosis in CRC cells. (a) The transcriptional level of hsa_circRNA_000166 was measured by qRT-PCR in both HCT116 and SW480 cells after transfection with siRNAs. (b) Cell proliferation assay showed that decreased hsa_circRNA_000166 expression limited the growth of HCT116 and SW480 cells after siRNA-1 or siRNA-2 transfection compared with the controls. (c) Clone formation assay demonstrated that the number of colonies was significantly decreased after siRNA-1 or siRNA-2 transfection compared with the controls in HCT116 and SW480 cells. (d) Flow cytometric analysis displayed that the number of apoptotic cells was noticeably increased in siRNA-1- or siRNA-2-transfected groups compared with the controls. The Student *t*-test was used for statistics.

According to the overexpression of hsa_circRNA_000166 in CRC cells, we knocked down the transcriptional level of hsa_circRNA_000166 using small interfering RNA (siRNA) to study the role of hsa_circRNA_000166 during CRC tumorigenesis. After transfection with siRNAs, we observed that the expression of hsa_circRNA_000166 was obviously decreased in siRNA-1- and siRNA-2-treated CRC cells and no significant changes in siRNA-3-treated CRC cells compared with controls by qRT-PCR analysis (Figure 3(a)), which certified that siRNA-1 and siRNA-2 could sufficiently knock down the transcriptional level of hsa_circRNA_000166. Subsequently, to demonstrate the function of hsa_circRNA_000166 in CRC cell growth, the CCK-8 assay was performed in HCT116 and SW480 cell lines after siRNA-1 or siRNA-2 transfection. We observed that the cell proliferation of CRC was limited in siRNA-1- or siRNA-2-treated groups compared with the controls in two CRC cell lines (Figure 3(b)). Meanwhile, we also conducted the colony formation assay in HCT116 and SW480 cell lines and revealed a notable decrease of colony number in siRNA-treated groups compared to the siRNA-NC groups in the two CRC cell lines (Figure 3(c)). Furthermore, to inspect the role of hsa_circRNA_000166 in CRC apoptosis, flow cytometry was utilized to calculate the apoptotic cells in both HCT116 and SW480 cell lines, and it was found that downregulation of hsa_circRNA_000166 resulted in the dramatic elevation of apoptosis rate in siRNA-1- or siRNA-2-treated groups rather than in controls (Figure 3(d)). In brief, our findings highlighted that downregulation of hsa_circRNA_000166 could suppress cell growth and enhance apoptosis in CRC cells.

### 3.3. hsa_circRNA_000166 Regulated CRC Progression by Inducing miR-326/LASP1 Axis

**Figure 4:**
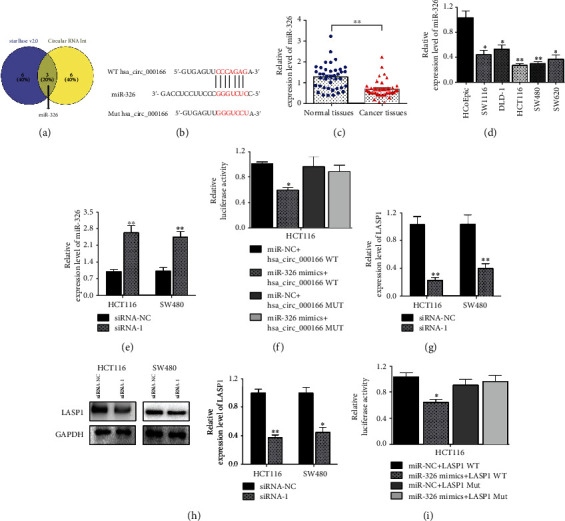
hsa_circRNA_000166 regulated CRC progression by targeting the miR-326/LASP1 axis. (a) The Venn analysis implied that 3 miRNAs, including miR-326, were involved in CRC. The blue represented 9 predicted miRNAs analyzed by starBase v2.0, and the yellow represented 9 predicted miRNAs analyzed by CircInteractome. (b) Binding site of hsa_circRNA_000166 in miR-326 was predicted by starBase. (c) qRT-PCR analysis showed that the transcriptional level of miR-326 was dramatically decreased in CRC tissues (*n* = 40) compared to the matched normal tissues (*n* = 40). (d) qRT-PCR analysis showed that miR-326 was notably downregulated in CRC cells compared with the normal colonic cells. (e) qRT-PCR assay displayed that miR-326 was significantly upregulated in siRNA-1-transfected groups compared with the controls in both HCT116 and SW480 cells. (f) Luciferase reporter assay indicated miR-326 dramatically repressed the WT hsa_circRNA_000166 luciferase activity but not Mut hsa_circRNA_000166 in HCT116 cells. (g, h) qRT-PCR and western blot assay showed that the transcriptional and translational levels of LASP1 were obviously downregulated in siRNA-1-transfected groups compared with the controls in both HCT116 and SW480 cells. (i) Luciferase reporter assay indicated miR-326 dramatically repressed the WT LASP1 luciferase activity but not Mut LASP1 in HCT116 cells. The Student *t*-test was used for statistics.

To elucidate the mechanism of hsa_circRNA_000166 in controlling CRC cell proliferation and apoptosis, we predicted that miR-326 was the candidate target of hsa_circRNA_000166 using the target prediction tool. Firstly, the Venn analysis between the predicted target miRNAs of hsa_circRNA_000166 and differential expressed miRNAs in CRC cells indicated that 3 miRNAs, containing miR-326, were involved in CRC using starBase and CircInteractome (Figure 4(a)). To certify whether miR-326 was a putative downstream target of hsa_circRNA_000166, we constructed plasmids with wild binding site or mutant binding site of hsa_circRNA_000166 into pGL3 vector with luciferase reporter gene (Figure 4(b)). Also, we detected the transcriptional level of miR-326 in CRC tissues and found its downregulation in CRC tissues compared with matched normal tissues (Figure 4(c)). Similarly, the expression of miR-326 was decreased in CRC cell lines than in the normal cell line HCoEpiC (Figure 4(d)). As shown in Figure 3, the inhibitory effect of siRNA-1 was better than siRNA-2; we select siRNA-1 for further investigation. After transfection with siRNA-1 or siRNA-NC, we observed that the transcriptional level of miR-326 was obviously upregulated in both HCT116 and SW480 cells (Figure 4(e)). Then, miR-NC and miR-326 I were separately cotransfected with hsa_circRNA_000166 WT and hsa_circRNA_000166 Mut luciferase reporter plasmid into HCT116 cells. Luciferase assay showed the relative luciferase activity in cells cotransfected with miR-326 I, and hsa_circRNA_000166 WT luciferase reporter plasmid was significantly decreased about 50% compared with the controls, while the relative luciferase activity of cells cotransfected with miR-326 I and hsa_circRNA_000166 Mut luciferase reporter plasmid has no obvious changes compared with the controls in HCT116 cells (Figure 4(f)). Recent studies reported that miR-326 could directly control the expression of LIM and SH3 protein 1 (LASP1) to suppress cell proliferation and activate apoptosis in hepatocellular carcinoma (HCC) [25]. Thus, we measured the transcriptional and translational levels of LASP1 after transfection with siRNA-1 and found a downregulation of LASP1 in both HCT116 and SW480 cells compared with the control groups using qRT-PCR and western blot assay (Figures 4(g) and 4(h)). Moreover, the miR-NC or miR-326 mimics were separately cotransfected with LASP1 WT and LASP1 Mut luciferase reporter plasmid into HCT116 cells. Luciferase assay showed the relative luciferase activity in cells cotransfected with miR-326 mimics, and LASP1 WT luciferase reporter plasmid was significantly decreased compared with the controls, while the relative luciferase activity of cells cotransfected with miR-326 mimics and LASP1 Mut luciferase reporter plasmid has no obvious changes compared with the controls in HCT116 cells (Figure 4(i)). The outcomes strongly indicated that hsa_circRNA_000166 might participate in CRC progression through targeting the miR-326/LASP1 pathway.

### 3.4. Inhibition of miR-326 or Overexpression of LASP1 Rescued the Phenotype Dominated by hsa_circRNA_000166

**Figure 5:**
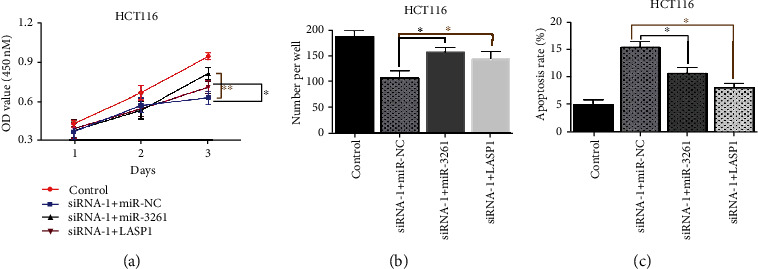
miR-326 downregulation or LASP1 upregulation rescued the phenotype dominated by hsa_circRNA_000166. (a, b) CCK-8 and colony formation assay demonstrated that cell codepletion of both siRNA-1 and miR-326 I or depletion of siRNA-1 while overexpression of LASP1 promoted cell growth compared with control groups in HCT116 cells. (c) The number of apoptotic cells in codepletion of both siRNA-1 and miR-326 I or depletion of siRNA-1 while overexpression of LASP1 groups was less than the number in control group HCT116 cells. The Student *t*-test was used for statistics.

To further confirm that miR-326 and LASP1 mediated the function of hsa_circRNA_000166 during CRC development, we conducted codepletion of both siRNA-1 and miR-326 I or depletion of siRNA-1 while there was overexpression of LASP1 and inspected the role of miR-326 and LASP1 in the regulation of hsa_circRNA_000166 in CRC development. CCK-8 and colony formation assay demonstrated that miR-326 downregulation or LASP1 overexpression in siRNA-1-transfected cells could restore cell growth compared with the controls in HCT116 cells (Figures 5(a) and 5(b)). Correspondingly, cells cotransfected with siRNA-1 and miR-326 I or siRNA-1 and LASP1 overexpression plasmid resulted in the significant decrease in the number of apoptotic cells compared with siRNA-1-transfected cells (Figure 5(c)). In conclusion, our studies identified hsa_circRNA_000166 was overexpressed in CRC cells and evidenced the potential function of hsa_circRNA_000166 in regulating the cell growth and apoptosis in CRC cells through directly interacting with the miR326/LASP1 axis. Therefore, hsa_circRNA_000166 might be a promising target in diagnostic and therapeutic application of CRC patient treatment.

## 4. Discussion

Colorectal cancer (CRC) is one of the solid tumors with a higher mortality among cancer-related deaths worldwide. Though advanced surgery technologies and medicine treatments have been applied in treating patients with CRC, the survival rate of patients with CRC is still poor. Therefore, there is an urgent demand for understanding the mechanisms underlying the development of CRC. In the past decades, circRNAs are discovered to be a subgroup of noncoding RNAs and play an essential role in regulating gene expression and function associated with cancers [15–19]. In this study, we identified that hsa_circRNA_000166 was one of the upregulated circRNAs with the highest expression level among all the upregulated circRNAs using bioinformatics analysis. Then, qRT-PCR assay was conducted to measure the expression of hsa_circRNA_000166 in CRC tissues and cell lines. We found that the transcriptional level of hsa_circRNA_000166 was notably increased, which highly correlated with poor 5-year survival rate of CRC patients. Next, we inhibited the expression of hsa_circRNA_000166 to perform further investigation. After downregulation of hsa_circRNA_000166, we observed that the cell growth and colony formation were limited and cell apoptosis was activated using corresponding assay. Similarly, Zhao and Dai have found that hsa_circRNA_000166 could promote cell proliferation, migration, and invasion by regulating miR-330-5p/ELK1 in colon cancers previously [20]. And our results were consistent with Zhao and Dai's findings. Collectively, these data strongly demonstrated that hsa_circRNA_000166 played an important role during CRC tumorigenesis.

Generally, circRNAs are sponging miRNAs to play its function in multiple biological processes, including tumorigenesis [21–23]. Previous studies have proved that multiple miRNAs mediated the function of circRNAs in CRC progression [24, 25]. Here, combined with bioinformatics analysis, we predicted that miR-326 might be a candidate target of hsa_circRNA_000166. Subsequently, luciferase assay was performed to confirm the direct interaction between hsa_circRNA_000166 and miR-326. Also, we measured the downregulation of miR-326 in CRC tissues and cell lines compared with the matched controls. Moreover, we revealed the LASP1, a downstream effector of miR-326 [26], was downregulated after siRNA-1 transfection compared to the controls, which suggested that the miR-326/LASP1 pathway was involved in the regulation of hsa_circRNA_000166 during CRC progression. Finally, we verified that downregulation of miR-326 could compromise the phenotype in siRNA-1-treated groups. Taken together, our findings evidenced that hsa_circRNA_000166 activated the cell growth and repressed apoptosis by sponging the miR-326/LASP1 axis during CRC tumorigenesis, which might be beneficial for diagnostic and therapeutic application in CRC treatment.

In this study, we used bioinformatics analysis to screen the GSE126094 dataset in CRC and identified that hsa_circRNA_000166 was the top 1 among all upregulated circRNAs. We further confirmed the overexpressions of hsa_circRNA_000166 in CRC tissues and cell lines and found that 5-year survival rate of CRC patients was highly related to the expression levels of hsa_circRNA_000166. Importantly, we confirmed that the miR-326/LASP1 pathway functions downstream of hsa_circRNA_000166 for the circRNA function during CRC progression. Together, our findings manifested that hsa_circRNA_000166 had a vital role in regulating CRC tumorigenesis, which implied that hsa_circRNA_000166 had a promising value in early diagnosis and prevention of CRC.

## Figures and Tables

**Table 1:** Gene-specific primers used for qRT-PCR.

Gene	Primer	Sequence 5′ to 3′
hsa_circRNA_000166	Forward	GCTTGGAACAGACTCACGGC
	Reverse	ATCTCCTGCCCAGTCTGACCT
miR-326	Forward	GGCGCCCAGAUAAUGCG
	Reverse	CGTGCAGGGTCCGAGGTC
LASP1	Forward	TGCGGCAAGATCGTGTATCC
	Reverse	GCAGTAGGGCTTCTTCTCGTAG
U6	Forward	TGCGGGTGCTCGCTTCGCAGC
	Reverse	CCAGTGCAGGGTCCGAGGT
GAPDH	Forward	ACACCCACTCCTCCACCTTT
	Reverse	TTACTCCTTGGAGGCCATGT

## Data Availability

The data used to support the findings of this study are available from the corresponding author upon request.
